# Absence of gastrointestinal infections in a cohort of patients with Zollinger-Ellison syndrome and other acid hypersecretors receiving long-term acid suppression with lansoprazole

**DOI:** 10.1186/1471-230X-8-18

**Published:** 2008-05-28

**Authors:** C Mel Wilcox, Toni Martin, Milind Phadnis, Jean Mohnen, Julie Worthington, Basil I Hirschowitz

**Affiliations:** 1 Department of Medicine, Division of Gastroenterology and Hepatology, University of Alabama at Birmingham, Birmingham, Alabama, USA; 2 School of Public Health, University of Alabama at Birmingham, Birmingham, Alabama, USA

## Abstract

**Background:**

The relationship between proton pump inhibitor therapy and other acid suppressing medications and the risk of gastrointestinal infections remains controversial.

**Methods:**

Patients enrolled in a long-term trial of lansoprazole for Zollinger-Ellison syndrome and other acid hypersecretory states had interval histories taken every six months regarding hospitalizations or other intercurrent medical conditions. All medications taken were also reviewed at each visit. In addition, available patients were specifically queried during the study period 2006–2007 regarding the development of any gastrointestinal infections, hospitalizations, and prescriptions for antibiotics.

**Results:**

Ninety patients were enrolled in our long-term study and 81 were available for review. The median basal gastric pH for the cohort after stabilization on therapy was 2.9 and ranged from 1.1 – 8.4 with a median pentagastrin stimulated gastric pH of 1.60 (range 1.0 – 8.2). No patient developed a clinically significant gastrointestinal infection during the study. The median patient years of follow-up were 6.25 years.

**Conclusion:**

In a cohort of patients with gastric acid hypersecretion in whom acid secretion status was monitored on lansoprazole, all were free of significant gastrointestinal infections on long-term follow-up.

**Trial registration:**

NCT00204373

## Background

Acid suppressive therapy with proton pump inhibitors is widespread, and their use has been associated with the development of gastrointestinal infections [1]. Recent studies raise concern that the use of these drugs may be linked to the development of *C. difficile *colitis [2-4]. Such observations have important implications given the extensive use of these drugs worldwide.

A cohort of patients with Zollinger-Ellison syndrome (ZES) and other hypersecretory conditions/pseudo ZES have been followed prospectively over a 18 year period as part of an ongoing study evaluating the efficacy and safety of lansoprazole therapy. In this protocol, patients undergo gastric acid analysis on a six monthly basis as well as evaluation for intercurrent illnesses or change in medical condition. Such a cohort provides a unique opportunity to evaluate the incidence of gastrointestinal infections and any relationship to the degree of gastric acid suppression over a prolonged period of continuous therapy.

## Methods

The protocol for patient evaluation and management has been previously published [5]. Briefly, patients with gastric acid hypersecretion were defined as a basal acid output > 15 mmol/h in patients without prior antrectomy or > 5 mmol/h after antrectomy. ZES was diagnosed by elevated fasting or secretin-stimulated gastrin levels and/or histologic identification of a gastrinoma. Sixty six patients had been treated for a median of 0.8 years, range 0.1–5, with omeprazole or other proton pump inhibitors before entering this study. Gastric acid analysis measuring basal and maximal stimulated acid secretion was performed at entry and at six month intervals following normalization of acid secretion with lansoprazole. Lansoprazole doses were individually titrated in each patient to maintain basal acid output (BAO) < 5 mmol/h a benchmark based on upper limit for healthy control subjects or < 2 mmol/h post-antrectomy.

Following initial stabilization of gastric acid secretion as noted above, patients were evaluated on a six monthly basis with history, physical examination, endoscopy with gastric biopsies, gastric analysis, serum gastrin, and other routine blood chemistry studies. Patients were also interviewed at each visit for any intercurrent illnesses, hospitalization/illness, and change in medications. All patients underwent interval physical examination and then underwent endoscopy by one of the authors throughout the study. In addition, over the last two years (2006–2007), when patients were seen in follow-up, they were specifically queried regarding the development of any gastrointestinal infections or other diarrheal illnesses while receiving lansoprazole with particular attention to episodes where the patient sought medical attention, had stool studies performed, or received antibiotics for therapy. All medical records for these hospitalizations were obtained whenever possible. In addition, patients were specifically queried about the use of antibiotics during the hospitalization or any received as an outpatient.

Fasting gastric analysis was performed every six months in the two hours before the next scheduled dose of lansoprazole. Each study comprised six consecutive ten-minute aspiration periods for one hour of basal secretion followed for one hour with stimulation by 6 ug/kg of pentagastrin (which accurately reflects maximal acid output with food [6]) or, more recently, as pentagastrin has become unavailable in the U.S., by modified sham feeding (vagal stimulation) [7]. In each sample, volume, pH, and titratable acid, were measured. The dose of lansoprazole was individually adjusted to maintain BAO at less than 5 mmol/hr, but to avoid excessive acid suppression. These data were used to determine whether, and to what extent, acid secretion was sufficiently suppressed to pH above 3.5, i.e., above the range of peptic activity [8]. We have further defined achlorhydria, for the purposes of this study, as a gastric pH above 5.0 – i.e., outside the range of any kind of gastric proteolysis, and bactericidal activity. The study has received approval from our institutional review board during its 18 years (IRB# F030107005). All patients gave written informed consent for the study.

## Results

Demographic information of the patients at study entry is shown in Table 1.

**Table 1:** Characteristic of the Study Group at Baseline

No.	81
Male, N (%)	51 (63.0%)
Caucasian, N (%)	60 (74.1%)
Age, mean (range)	51.6 ± 1.5 (22, 88)
ZES, N (%)	63 (76.8%)
MEN – 1, N (%)	6 (7.3%)
Prior antrectomy, N (%)	10 (12.3%)
Serum gastrin concentration, median (range)	
ZE	493 (38, 17,491)
Non ZE*	73 (30, 262)
BAO mEq/L, median (range)	22.3 (1.5, 96.5)
MAO mEq/L, median (range)	47.9 (7.2, 121.3)
H. pylori positive, N (%)	52 (64.2%)

* Non ZE – non ZE acid hypersecretors

Lansoprazole was given for 2 – 207 months with a median of seventy five months, representing a total of 45,862 patient-months of treatment. For the cohort, following stabilization, the median BAO was 2.4 +/- 0.3. Likewise, following stabilization, the median PAO was appropriately suppressed (Table 2). After lansoprazole treatment, the median gastric pH was 2.9 at basal level and 1.6 after stimulation with either pentagastrin or sham feeding. Optimized effective doses of lansoprazole varied from 15 mg every other day to 450 mg per day with the median of 75 mg per day.

**Table 2:** Duration on PPI, acid outputs, and pH before and during treatment with lansoprazole

N = 81	**Before Lansoprazole**	**After Lansoprazole**
**Duration on PPI **(Years)	1.2 ± 0.2	7.1 ± 0.6
Median (range)	0.8 (0.1, 5.0)	6.25 (0,16)
		
**Acid output**		
**BAO **(mmol/h)	25.1 ± 1.9	2.4 ± 0.3
**PAO **(mmol/h)	51.0 ± 2.9	10.4 ± 1.0
		
**pH **Median (range)		
Basal pH	1.2 (1.0, 8.2)	2.9 (1.1, 8.1)
Stimulated pH	1.1 (1.0, 8.0)	1.6 (1.0, 8.2)

During this study, 53 patients (65%) required hospitalization for a variety of reasons (Table 3). Some patients had more than one admission. Based upon the reason for hospitalization, review of hospital records, and discussion with the patient, antibiotics were provided during the hospitalization in 38 patients (72%). Additional antibiotic use as an outpatient was reported in 42 patients (52%). Antibiotic classes used included pencicillin/cephalosporin (40%), fluroquinolones (20%), metronidazole (19%), sulfa macrolide (15%), sulfa (2%), and other (10%). Some patients took antibiotics of different classes during the study period. Only one patient reported any significant gastroenteritis during the study period. This patient following travel outside the U.S. experienced several days of watery diarrhea which was apparently self limited. He was evaluated by a physician and his stool tests were negative for any infection. No patient was diagnosed with *C. difficile *colitis or bacterial gastroenteritis. Recall for the specific antibiotics taken, however, was infrequent.

**Table 3:** Broad indications for hospitalization

	N (%)
Surgery	12 (23)
Chest pain/heart disease	11 (21)
Infection	9 (17)
Nausea/vomiting Central nervous system disorder	7 (13) 5 (9)
Other*	13 (25)

* Other – lung disease (4), nephrolithiasis (3), abnormal liver tests (3), anemia (1), gout (1), dehydration (1)

Some patients had more than one hospitalization.

## Discussion

Long-term follow-up in a large cohort of acid hypersecretors with known gastric acid secretory status while on lansoprazole therapy failed to identify any evidence of significant gastrointestinal infections. With our extensive years of treatment and follow-up, only one patient sought medical care for a reported gastroenteritis and there were no apparent hospitalizations for gastrointestinal infection during this study.

The relationship between proton pump inhibitor therapy and other acid suppressing medications and the risk of gastrointestinal infections remains controversial. Since the initial use of histamine-II receptor antagonists in 1973, there has been concern about the possibility of gastrointestinal infections with the use of these agents, presumably related to fecal-oral transmission of bacteria, which were not killed by an acid milieu in the stomach. More recent epidemiological studies have focused on drugs inhibiting gastric acid secretion and the development of *C. difficile *colitis. For example, in a prospective case controlled study of 155 consecutive inpatients with *C. difficile *diarrhea, 92% had received acid suppression compared with 50% of controls [4]. Logistic regression also identified acid suppression (odds ratio 1.9, 94% CI 1.1, 3.29) as a risk factor. Large epidemiological studies also support an association [3]. However, other studies, primarily retrospective case control studies, have failed to show an association [9,10]. A population based case control study from Canada where patients were matched based on age and gender, as well as antibiotic, found no association between PPI use and hospitalization for *C. difficile *disease [11]. One might anticipate a heightened risk of infections in patients with pernicious anemia, but this has been surprisingly not well documented in any large case series.

In vitro studies show that normal gastric juice with a pH < 4 is bactericidal while achlorhydric gastric juice is not [12]. At a gastric pH < 3, bacteria instilled in the normal human stomach are killed, but may remain viable for an hour in the achlorhydric stomach [13]. In healthy volunteers, the use of proton pump inhibitor therapy consistently elevates gastric pH. The 24 hour intragastric pH may remain > 4 ranging from 40%- over 65% of the monitoring period in patients enrolled in short term studies [14-17]. Gastric acid analysis in our acid hypersecretor cohort showed consistently blunted acid secretion yet with sham feeding stimulation, the basal gastric pH was still < 3.5 in 67%, and with pentagastrin (the equivalent of food) in 84%.

Our study has several limitations which should be recognized. Firstly, while patients were seen on an every six month basis and were queried regarding hospitalizations, need for surgery, and intercurrent medication use, patients were not prospectively queried at each point in time regarding the development of any gastrointestinal infections. Secondly, while antibiotic use was assessed, most patients could not recall nor was there documentation in many cases of the specific antibiotic and dose used, but only the class of drug. Thirdly, querying patients regarding any gastrointestinal infection in some patient's years after study enrollment could lead to recall bias. Nevertheless, all hospitalizations were documented. Fourth, depending on the absolute risk of infection with these infections, our sample size may be inadequate, yet our long term follow-up assessed by patient years is extensive. One might have expected more reported episodes of diarrheal illness but we focused on those more severe where health care input was sought. Lastly, our study population consists of acid hypersecretors which may be different than those with underlying normal acid secretion who may have less acid secretion while on PPI therapy.

## Conclusion

In conclusion, our large prospective cohort study of patients with acid hypersecretory states including ZES undergoing twice yearly evaluations and gastric acid analysis failed to identify patients experiencing gastrointestinal infections at a median follow-up of greater than six years. Further long-term follow-up is important to assess for any infection risk in our cohort.

## List of Abbreviations used

ZES: Zollinger Ellison Syndrome; BAO: basal acid output; PAO: peak acid output.

## Competing interests

The study was funded by TAP Pharmaceuticals.

## Authors' contributions

CMW responsible for study design and drafted and helped prepare the manuscript. BIH responsible for study design and drafted and helped prepare the manuscript. JW was responsible for data analysis. MP was responsible for data analysis. TM was responsible for data collection. JM was responsible for data collection. All of the authors read and approved the final manuscript.

## Pre-publication history

The pre-publication history for this paper can be accessed here:


